# Cytotoxic Effects of Curcumin in Human Retinal Pigment Epithelial Cells

**DOI:** 10.1371/journal.pone.0059603

**Published:** 2013-03-26

**Authors:** Margrit Hollborn, Rui Chen, Peter Wiedemann, Andreas Reichenbach, Andreas Bringmann, Leon Kohen

**Affiliations:** 1 Department of Ophthalmology and Eye Hospital, University of Leipzig, Leipzig, Germany; 2 Paul Flechsig Institute of Brain Research, University of Leipzig, Leipzig, Germany; 3 Helios Klinikum Aue, Aue, Germany; University of Helsinki, Finland

## Abstract

**Backround:**

Curcumin from turmeric is an ingredient in curry powders. Due to its antiinflammatory, antioxidant and anticarcinogenic effects, curcumin is a promising drug for the treatment of cancer and retinal diseases. We investigated whether curcumin alters the viability and physiological properties of human retinal pigment epithelial (RPE) cells *in vitro*.

**Methodology/Principal Findings:**

Cellular proliferation was investigated with a bromodeoxy-uridine immunoassay, and chemotaxis was investigated with a Boyden chamber assay. Cell viability was determined by trypan blue exclusion. Apoptosis and necrosis rates were determined with a DNA fragmentation ELISA. Gene expression was determined by real-time PCR, and secretion of VEGF and bFGF was examined with ELISA. The phosphorylation level of proteins was revealed by Western blotting. The proliferation of RPE cells was slightly increased by curcumin at 10 µM and strongly reduced by curcumin above 50 µM. Curcumin at 50 µM increased slightly the chemotaxis of the cells. Curcumin reduced the expression and secretion of VEGF under control conditions and abolished the VEGF secretion induced by PDGF and chemical hypoxia. Whereas low concentrations of curcumin stimulated the expression of bFGF and HGF, high concentrations caused downregulation of both factors. Curcumin decreased dose-dependently the viability of RPE cells via induction of early necrosis (above 10 µM) and delayed apoptosis (above 1 µM). The cytotoxic effect of curcumin involved activation of caspase-3 and calpain, intracellular calcium signaling, mitochondrial permeability, oxidative stress, increased phosphorylation of p38 MAPK and decreased phosphorylation of Akt protein.

**Conclusion:**

It is concluded that curcumin at concentrations described to be effective in the treatment of tumor cells and in inhibiting death of retinal neurons (∼10 µM) has adverse effects on RPE cells. It is suggested that, during the intake of curcumin as concomitant therapy of cancer or in the treatment of eye diseases, retinal function should be monitored carefully.

## Introduction

The natural phenolic compound curcumin (diferuloylmethane), the yellow pigment of turmeric and an ingredient in curry powders, has a long history of use in traditional Asian medicine for a wide variety of disorders. Curcumin has been shown to have antiinflammatory, antioxidant, and antiproliferative effects in various cell systems [1], [2]. In addition, curcumin has antiviral effects, inhibits the proliferation of bacteria and fungi [3]–[6], and induces immunosupression in subjects with renal transplants [7]. Curcumin is recognized as a promising anticancer drug and is believed to be helpful as concomitant therapy of a variety of diseases associated with chronic inflammation and as adjuvant immunosuppressant [1], [8], [9]. Curcumin influences multiple intracellular signaling pathways and has both antioxidant and prooxidant effects in dependence on the concentration of the compound [10]–[13]. It induces apoptosis of cancer cells by activation of procaspases and the release of cytochrome c from mitochondria [14]–[16]. Both antioxidant and prooxidant activities were shown to be involved in the anticancer activity of curcumin [10]–[13]. However, it is conceivable that cytotoxic effects of curcumin may also concern non-transformed cells. Indeed, it has been found that curcumin may cause toxicity to non-transformed cells under some circumstances [9], [17], [18].

Elevated oxidative stress contributes to the pathogenesis of various blinding retinal diseases including diabetic retinopathy, retinitis pigmentosa, and age-related macular degeneration [19]–[24]. Antioxidant nutrients may decrease the risk of the development of age-related macular degeneration [25]–[27]. Based upon data obtained in animal models of retinopathies and cultured retinal cells, it has been suggested that curcumin could have potential benefits in inhibiting the development of diabetic retinopathy, age-related macular degeneration, and retinitis pigmentosa [28]–[30]. Dietary curcumin reduced oxidative changes and inhibited the elevations of interleukin-1ß, tumor necrosis factor (TNF)-α, and vascular endothelial growth factor (VEGF) in the retina of hyperglycemic rats [28], [31], [32]. Dietary curcumin also inhibited the upregulation of inflammatory genes in a rat model of light-induced retinal degeneration, and protected retinal cells from oxidative cell death [29]. It protected photoreceptors from degeneration in a transgenic rat model of retinitis pigmentosa [30], prevented staurosporine-induced death of retinal ganglion and amacrine cells in the murine retina [33], and inhibited neuronal apoptosis and microvessel degeneration in experimental ischemia-reperfusion injury of the retina [34]. However, it has also been shown that curcumin induces apoptosis in human retinal endothelial cells [35].

Little is known regarding the effects of curcumin in retinal pigment epithelial (RPE) cells. RPE cells play crucial roles in protecting the outer retina from photooxidative stress, in the digestion of shed photoreceptor outer segments which contain oxidized lipids, and in inhibition of retinal edema and neovascularization [36]. Dysfunction and degeneration of RPE cells are crucially involved in the pathogenesis of age-related macular degeneration [37], [38]. The dry form of this blinding disease is characterized by (among others) the presence of lipofuscin within the RPE and drusen beneath the RPE which both contain photoreceptor-derived oxidized lipids, as well as by RPE cell death (geographic atrophy), while the hallmarks of the wet form are choroidal neovascularization and subretinal edema induced by outer retinal hypoxia [37], [38]. VEGF is the major hypoxia-induced angiogenic factor that promotes retinal neovascularization and edema [39]–[41]. RPE cells are an important source of VEGF in the retina [42]. In addition to VEGF, basic fibroblast growth factor (bFGF) is a mediator of retinal neovascular diseases such as diabetic retinopathy and the wet form of age-dependent macular degeneration [43]. Cell scattering induced by hepatocyte growth factor (HGF) is a precondition of RPE cell migration and proliferation involved in choroidal neovascularization and proliferative retinopathies [44], [45]. It has been shown recently that curcumin reduces the viability of RPE cells by inducing caspase activation [46]. In the present study, we examined the mechanisms of the toxic effect of curcumin in human RPE cells and determined the effects of curcumin on the expression and secretion of angiogenic factors from the cells.

## Materials and Methods

### Ethics Statement

The study followed the tenets of Declaration of Helsinki for the use of human subjects. The use of human material was approved by the Ethics Committee of the University of Leipzig (approval #745, 07/25/2011). Tissues were obtained with the written informed consent from relatives of all donors.

### Materials

All tissue culture components and solutions were purchased from Gibco BRL (Paisley, UK). Recombinant human platelet-derived growth factor (PDGF)-BB was purchased from R&D Systems (Minneapolis, MN). The peptides Ac-DEVD-CHO (asp-glu-val-asp) and Ac-IETD-CHO (ile-glu-thr-asp) were from Enzo Life Science (Lörrach, Germany). PD150606, bis-(o-aminophenoxy)ethane-*N*,*N*,*N′*,*N′*-tetra-acetic acid acetoxymethyl ester (BAPTA-AM), and cyclosporin A were from Calbiochem (Bad Soden, Germany). All other substances and agents used were from Sigma-Aldrich (Taufkirchen, Germany), unless stated otherwise. Lipophilic substances were dissolved in dimethylsulfoxide; the final dilution of dimethylsulfoxide used in the experiments was 1∶1000. Curcumin was dissolved in ethanol (0.2%). The following antibodies were used: neutralizing rabbit anti-human TNFα (Abcam, Cambridge, UK; 20 µg/ml), a rabbit anti-GAPDH (New England Biolabs, Frankfurt/M., Germany; 1∶2000), a rabbit anti-human extracellular signal-regulated kinases 1 and 2 (ERK1/2; p44/p42; New England Biolabs; 1∶1000), a rabbit anti-phosphorylated ERK1/2 (New England Biolabs; 1∶1000), a rabbit anti-human Akt (New England Biolabs; 1∶1000), a rabbit anti-human phosphorylated Akt (New England Biolabs; 1∶1000), a rabbit anti-human p38 mitogen-activated protein kinase (p38 MAPK; New England Biolabs; 1∶1000), a rabbit anti-human phosphorylated p38 MAPK (New England Biolabs; 1∶750), a rabbit anti-human stress-activated protein kinase (SAPK)/c-Jun N-terminal kinase (JNK) (New England Biolabs; 1∶1000), a rabbit anti-human phosphorylated-SAPK/JNK (New England Biolabs; 1∶1000), a rabbit anti-human heat shock protein (HSP) 70 (New England Biolabs; 1∶750), and anti-rabbit IgG conjugated with alkaline phosphatase (Chemicon, Hofheim, Germany; 1∶3000).

### Cell Culture

Human RPE cells were obtained from several donors within 48 hours of death, and were prepared and cultured as following. After removing the vitreous and the retina, the RPE cells were mechanically harvested, separated by digestion with 0.05% trypsin and 0.02% EDTA and washed two times with phosphate-buffered saline. The cells were suspended in complete Ham F-10 medium containing 10% fetal bovine serum, glutamax II, and penicillin/streptomycin, and were cultured in tissue culture flasks (Greiner, Nürtingen, Germany) in 95% air/5% CO_2_ at 37°C. Cells of passages 3 to 5 were used. The epithelial nature of the RPE cells was routinely identified by immunocytochemistry using the monoclonal antibodies AE1 (recognizing most of the acidic type I keratins) and AE3 (recognizing most of the basic type II keratins), both from Chemicon. To test the substances, near-confluent cultures were growth arrested in medium without serum over night, and subsequently, media without serum (PCR, western blotting) or containing 0.5% serum with and without test substances were added.

### DNA Synthesis Rate

The proliferation rate of RPE cells was determined by measuring the incorporation of bromodeoxyuridine (BrdU) into the genomic DNA. The cells were seeded at 3×10^3^ cells per well in 96-well microtiter plates (Greiner), and were allowed to attach for 48–72 h (∼90% confluency). Thereafter, the cells were growth arrested in medium without serum for 5 h. Subsequently, medium containing 0.5% serum without and with PDGF (10 ng/ml) and curcumin at different concentrations was added for another 24 h. Vehicle control was made with ethanol (0.2%). BrdU incorporation was determined by using the Cell Proliferation ELISA BrdU Kit (Roche, Mannheim, Germany). BrdU (10 µM) was added to the culture medium 5 h before fixation.

### Chemotaxis

Chemotaxis was determined with a modified Boyden chamber assay. Suspensions of RPE cells (100 µl; 5×10^5^ cells/ml; medium containing 0.5% serum) were seeded onto polyethylene terephthalate filters (diameter 6.4 mm, pore size 8 µm; Becton Dickinson, Heidelberg, Germany) coated with fibronectin (50 µg/ml) and gelatin (0.5 mg/ml). Within 16 h after seeding, the cells attached to the filter and formed a semiconfluent monolayer. Thereafter the medium was changed into medium without additives in the upper well and medium containing PDGF (10 ng/ml) and curcumin at different concentrations in the lower well. Vehicle control was made with ethanol (0.2%). Data obtained in cultures which were pretreated with curcumin for 30 min and which were treated with curcumin simultaneously with PDGF were not significantly different (not shown). After incubation for 6 h, the inserts were washed with buffered saline, fixed with Karnofskỳs reagent, and stained with hematoxylin. Nonmigrated cells were removed from the filters by gentle scrubbing with a cotton swab. The migrated cells were counted, and the results were expressed relative to the cell migration rate in the absence of test substances.

### Cell Viability

The number of viable cells was determined by trypan blue exclusion. The cells were seeded at 5×10^4^ cells per well in 6-well plates. After reaching a confluency of ∼90%, the cells were cultured in serum-free medium for 16 h and then stimulated with curcumin or triamcinolone acetonide at different concentrations for 24 h. After trypsinization, the cells were stained with trypan blue (0.4%), and the number of viable (non-stained) and dead (stained) cells were determined using a hemocytometer.

### DNA Fragmentation

The Cell DNA Fragmentation ELISA (Roche) was used to determine whether the cells undergo apoptosis or necrosis in the absence and presence of curcumin at different concentrations. Vehicle control was made with ethanol (0.2%). The cells were seeded at 3×10^3^ cells per well in 96-well plates, and were cultured until confluency was reached. After changing the culture media, the cells were prelabeled with BrdU for 16 h and then incubated in the absence or presence of curcumin in F-10/0.5% fetal calf serum for 6 and 24 h, respectively. Necrosis was determined by analyzing the BrdU-labeled DNA fragments in the cell-free culture supernatants, and apoptosis was determined by using the cytoplasmic lysates of the cells.

The mechanisms of curcumin-induced DNA fragmentation in the cultured media were determined after a 6 h stimulation of the cultures with curcumin (50 µM) in the absence and presence of the following agents: Ac-DEVD-CHO (100 µM), Ac-IETD-CHO (100 µM), PD150606 (100 µM), BAPTA-AM (100 µM), dithiothreitol (3 mM), cyclosporin A (1 µM), and a neutralizing anti-TNFα antibody (20 µg/ml).

### mRNA Expression

The gene expression of VEGF-A, bFGF, HGF, caspase-3, and Smac was determined in the absence and presence of curcumin at different concentrations after 2, 6, and 24 h. Vehicle control was made with ethanol (0.2%).

### Total RNA Isolation

Total RNA was extracted from cultured cells by using the RNeasy Mini Kit (Qiagen, Hilden, Germany). The quality of the RNA was analyzed by agarose gel electrophoresis. The A_260_/A_280_ ratio of optical density was measured using the NANODROP 1000 device (Peqlab, Erlangen, Germany), and was between 1.9 and 2.1 for all RNA samples, indicating sufficient quality.

Real-time RT-PCR After treatment with DNase I (Roche), cDNA was synthesized from 1 µg of total RNA using the RevertAid H Minus First Strand cDNA Synthesis Kit (Fermentas, St. Leon-Roth, Germany). For subsequent PCR amplification, the cDNA was diluted by addition of 20 µl RNase free water. Semi-quantitative real-time RT-PCR was performed with the Single-Color Real-Time PCR Detection System (BioRad, Munich, Germany) using the primer pairs described in Table 1. The PCR solution contained 1 µl cDNA, specific primer set (0.25 µM each) and 10 µl of 10 µl of iQ SYBR Green Supermix (BioRad) in a final volume of 20 µl. The following conditions were used: initial denaturation and enzyme activation (one cycle at 95°C for 3 min); denaturation, amplification and quantification, 45 cycles at 95°C for 30 s, 58°C for 20 s, and 72°C for 45 s. This was followed by a melt curve analysis (81 cycles) to determine the product specificity where the temperature was gradually increased (0.5°C/cycle) from 55°C to 95°C. The amplified samples were analyzed by standard agarose gel electrophoresis. The mRNA expression was normalized to the level of *ACTB* expression. The changes in mRNA expression were calculated according to the 2^−ΔΔCT^ method (CT, cycle threshold), with ΔCT = CT_target gene_−CT_actb_ and ΔΔCT = ΔCT_treatment_−ΔCT_control_.

**Table 1:** Primer pairs used for PCR experiments. s, sense. as, anti-sense.

Gene and Accession	Primer sequence (5′→3′)	Amplicon (bp)
ACTB	s ATGGCCACGGCTGCTTCCAGC	237
NM_001101	as CATGGTGGTGCCGCCAGACAG	
VEGFA	s CCTGGTGGACATCTTCCAGGAGTA	407; 347; 275
NM_001025370	as CTCACCGCCTCGGCTTGTCACA	
BFGF	s AGAGCGACCCTCACATCAAG	234
NM_002006	as ACTGCCCAGTTCGTTTCAGT	
HGF	s GGCTGGGGCTACACTGGATTG	179
NM_000601	as CCACCATAATCCCCCTCACAT	
CASP3	s ACTGGAAAACCCAAACTTTTCAT	245
NM_004346	as ATAAATTCAAGCTTGTCGGCATA	
CASP8	s CATCCAGTCACTTTGCCAGA	128
NM_033356.3	as GCATCTGTTTCCCCATGTTT	
BAX	s CAAGAAGCTGAGCGAGTGTCT	152
NM_004324	as AGTTGAAGTTGCCGTCAGAAA	
SMAC	s ACTTGGGAAAATGAATTCAGAGG	163
NM_138929.3	as AGTTTGATATGCAGCTTCTGCT	
HSP70	s GGAGGAGTTCAAGAGAAAACACA	157
NM_005345.5	as GTAGAAGTCGATGCCCTCAAAC	

### ELISA

The cells were cultured at 3×10^3^ cells per well in 96-well plates (100 µl culture medium per well). At a confluency of ∼90%, the cells were cultured in serum-free medium for 16 h. Subsequently, the culture medium was changed, and the cells were stimulated with PDGF (10 ng/ml) and CoCl_2_ (150 µM), respectively, in the absence and presence of curcumin at different concentrations. Vehicle control was made with ethanol (0.2%). The supernatants were collected after 6 h, and the levels of VEGF-A_165_ and bFGF, respectively, in the cultured media (200 µl for VEGF; 100 µl for bFGF) were determined by ELISA (R&D Systems).

### Western Blotting

The cells were seeded at 1×10^5^ cells per well in 6 well plates in 1.5 ml complete medium, and were allowed to growth up to a confluency of ∼90%. After growth arrest for 16 h, the cells were treated with PDGF (10 ng/ml) or curcumin at different concentrations (in the absence and presence of 10 µM SB203580) for 15 min. Cultures were pretreated with SB203580 for 30 min. A HSP70 positive control was made by incubation of the cells at 42°C (water bath) for 1 h (after sealing of the culture plates with parafilm) followed by incubation at 37°C for 3 h, as previously described [47]. Vehicle control was made with ethanol (0.2%). After the treatment of the cultures, the medium was removed, the cells were washed twice with prechilled phosphate-buffered saline (pH 7.4; Invitrogen, Paisley, UK), and the monolayer was scraped into 150 µl lysis buffer (Mammalian Cell Lysis-1 Kit; Sigma). The total cell lysates were centrifuged at 10,000×*g* for 10 min, and the supernatants were analyzed by immunoblots. Equal amounts of protein (30 µg) were separated by 10% SDS-polyacrylamide gel electrophoresis. Immunoblots were probed with primary and secondary antibodies, and immunoreactive bands were visualized using 5-bromo-4-chloro-3-indolyl phosphate/nitro blue tetrazolium.

### Statistics

For each test, at least 3 independent experiments were carried out in triplicate using cells from different donors. Data are expressed as means ± SEM; statistical significance (Mann-Whitney *U* test) was accepted at *P*<0.05.

## Results

### Expression and Secretion of Cytokines

**Figure 1:**
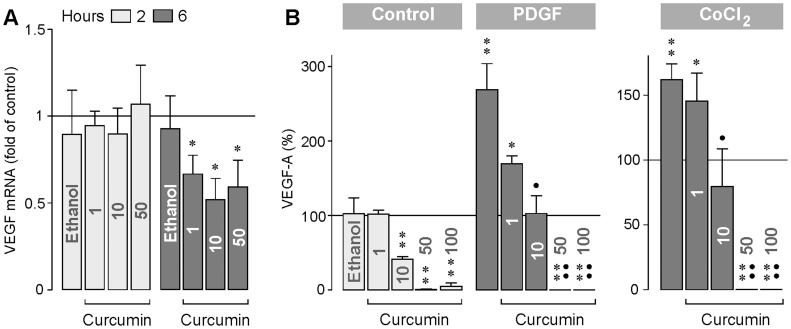
Curcumin inhibits expression and secretion of VEGF. **A.** Concentration-dependent effect of curcumin on the gene expression of VEGF-A. The mRNA level was determined by real-time RT-PCR after stimulation of the cells with curcumin for 2 and 6 h, respectively. **B.** Concentration-dependent effect of curcumin on the secretion of VEGF from RPE cells. The effects were determined in the absence (control) and presence of PDGF (10 ng/ml) and CoCl_2_ (150 µM), respectively, for 6 h. The level of VEGF-A_165_ in the cultured media was determined by ELISA. The concentration of curcumin (in µM) is given in the bars. The vehicle control was made with ethanol (0.2%). Data are means ± SEM of 3–6 independent experiments carried out in triplicate using cells from different donors. Significant difference *vs*. untreated control: *P<0.05; **P<0.01. Significant difference *vs*. PDGF and CoCl_2_, respectively: ^•^ *P*<0.05; ^••^ *P*<0.01.

We determined whether curcumin may inhibit the expression and secretion of angiogenic cytokines in cultured human RPE cells. As shown in Figure 1A, treatment of the cultures with curcumin for 2 h did not alter the gene expression of VEGF compared to unstimulated control. However, treatment of the cultures with curcumin for 6 h resulted in a significant (*P*<0.05) decrease in the gene expression of VEGF compared to control. The secretion of VEGF protein from RPE cells was previously described to be stimulated by PDGF and under chemical hypoxia conditions induced by addition of CoCl_2_ to the culture medium [48], [49]. Curcumin had a dose-dependent inhibitory effect on the secretion of VEGF from RPE cells. Curcumin at 10 µM decreased significantly the secretion of VEGF under unstimulated control conditions, during stimulation of the cultures with PDGF, and under chemical hypoxia conditions induced by CoCl_2_ (Fig. 1B). Curcumin at 50 and 100 µM fully inhibited the secretion of VEGF from RPE cells (Fig. 1B).

**Figure 2:**
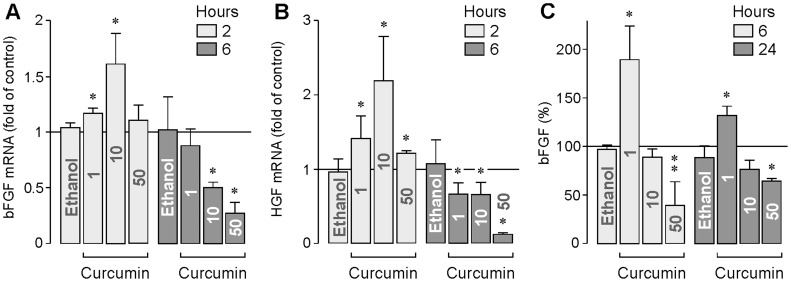
Effects of curcumin on the gene expression of bFGF (A) and HGF (B). The mRNA levels were determined by real-time RT-PCR after stimulation of the cells with curcumin for 2 and 6 h, respectively. **C.** Effect of curcumin on the secretion of bFGF from RPE cells. The level of bFGF in the cultured media was determined by ELISA after stimulation of the cells with curcumin for 6 and 24 h, respectively. The concentration of curcumin (in µM) is given in the bars. The vehicle control was made with ethanol (0.2%). Data are means ± SEM of 4–5 independent experiments carried out in triplicate using cells from different donors. Significant difference *vs*. untreated control: *P<0.05; **P<0.01.

Curcumin displayed also dose- and time-dependent effects on the expression of bFGF and HGF. Whereas curcumin at lower concentrations significantly (*P*<0.05) increased the gene expression of bFGF (Fig. 2A) and HGF (Fig. 2B) after 2 h of stimulation, curcumin at higher concentrations decreased the gene expression of both factors after 6 h of stimulation (Fig. 2A, B). The secretion of bFGF from RPE cells was stimulated in the presence of curcumin at 1 µM, and decreased in the presence of curcumin at 50 µM (Fig. 2C).

### Proliferation and Chemotaxis

**Figure 3:**
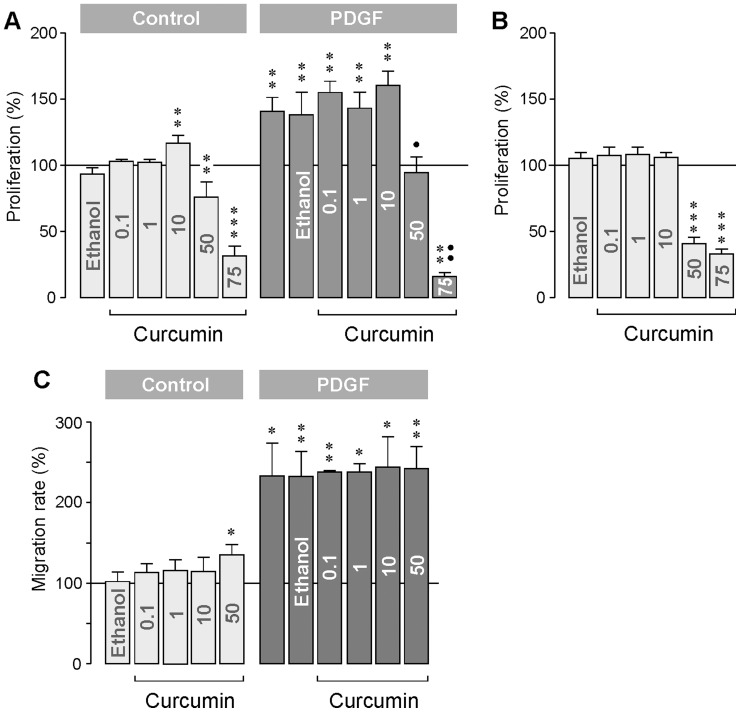
Concentration-dependent effects of curcumin on the proliferation (A, B) and chemotaxis (C) of RPE cells. The rate of BrdU incorporation was measured after a 24 h (**A**) and 96 h (**B**) incubation with the agents, respectively. The concentration of curcumin (in µM) is given in the bars. The effects were determined in the absence (control) and presence of PDGF (10 ng/ml). The vehicle control was made with ethanol (0.2%). Data are means ± SEM of 4–8 independent experiments carried out in triplicate using cells from different donors, and are expressed in percent of untreated control (100%). Significant difference *vs*. untreated control: *P<0.05; **P<0.01; ***P<0.001. Significant difference *vs*. PDGF control: ^•^ *P*<0.05; ^••^ *P*<0.01.

We measured the proliferation and chemotaxis of cultured cells in the absence and presence of PDGF, a known mitogen and motogen of RPE cells [48]. As shown in Figure 3A, curcumin altered dose-dependently the proliferation of RPE cells. Curcumin at 10 µM stimulated the proliferation of the cells, whereas curcumin at 50 and 75 µM decreased the proliferation rate. The inhibitory effect of high-dose curcumin on cellular proliferation was found after both 24 h (Fig. 3A) and 96 h (Fig. 3B) of incubation. The curcumin-induced decrease in RPE cell proliferation was also observed under PDGF-stimulated conditions (Fig. 3A). Curcumin at concentrations between 0.1 and 10 µM did not alter the chemotaxis of RPE cells under both control and PDGF-stimulated conditions (Fig. 3C). Curcumin at 50 µM increased significantly (*P*<0.05) the chemotaxis under control conditions (Fig. 3C).

### Cell Viability

**Figure 4:**
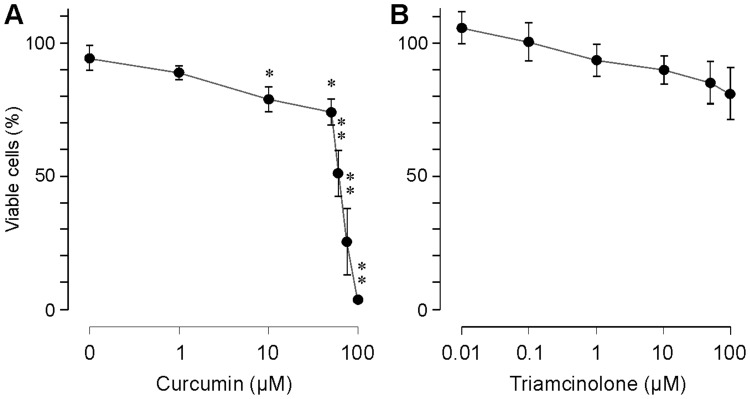
Concentration-dependent effects of curcumin (A) and triamcinolone acetonide (B) on the viability of RPE cells. The percentage of viable cells was evaluated 24 h after addition of the test substances to the culture medium. Data are means ± SEM of 5–7 independent experiments carried out in triplicate using cells from different donors, and are expressed in percent of untreated control (100%). Significant difference *vs*. control: *P<0.05; **P<0.01.

We found that curcumin at concentrations above 10 µM decreased the proliferation of RPE cells (Fig. 3A, B). To determine whether this effect was caused by a decrease in the viability of the cells, we stained the cells with trypan blue and counted the living and dead cells. As shown in Figure 4A, curcumin decreased slightly the number of viable cells at concentrations between 1 and 50 µM. At higher concentrations (75 and 100 µM), curcumin strongly decreased the viability of the cells; almost no viable cells were found after the treatment of the cultures with curcumin at 100 µM (Fig. 4A). We compared the effect of curcumin on cell viability with the effect of triamcinolone acetonide, an antiinflammatory steroid clinically used in the treatment of inflammatory and ischemic retinopathies associated with edema [50]. Though triamcinolone also decreased slightly the viability of the cells at higher concentrations, this effect was not significant (Fig. 4B).

### DNA Fragmentation

**Figure 5:**
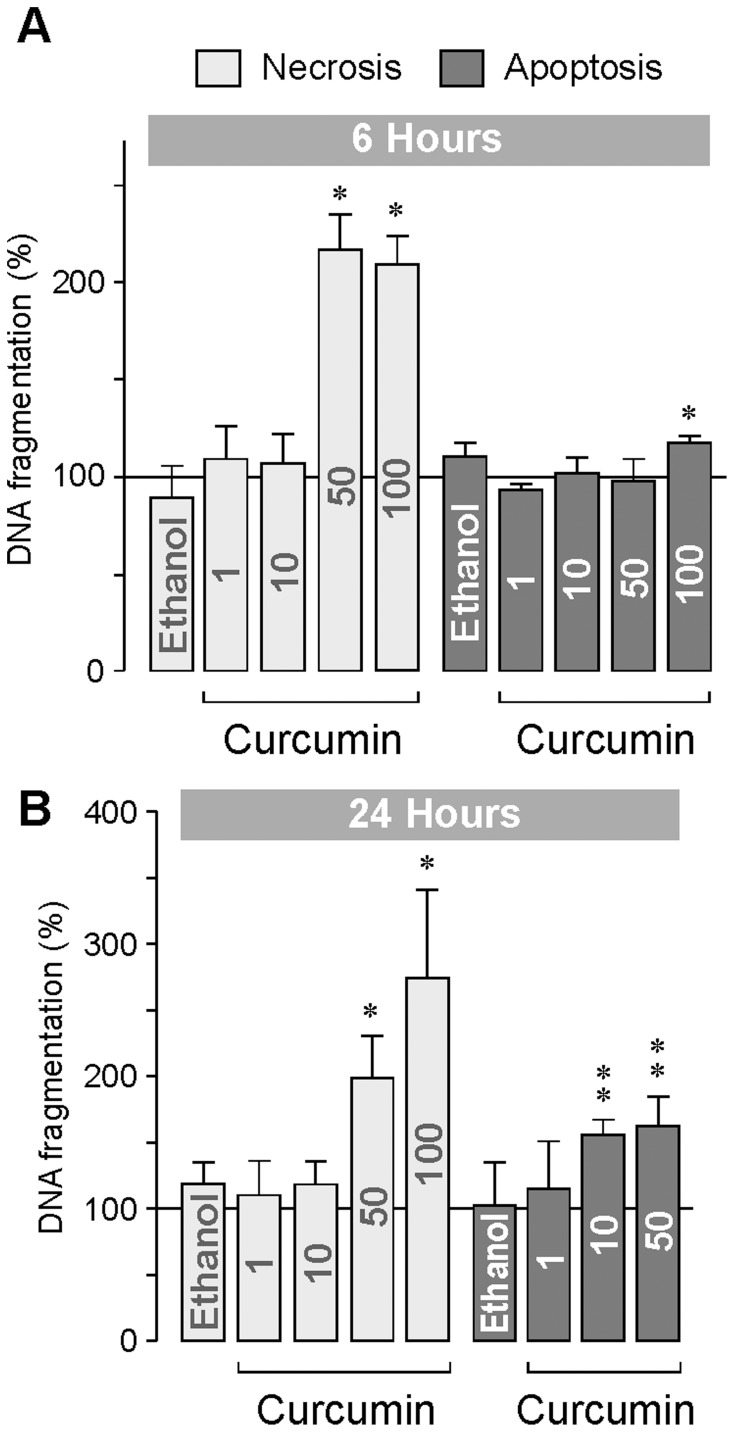
Concentration- and time-dependent effect of curcumin on the rate of internucleosomal DNA fragmentation of RPE cells. The rate of DNA fragmentation was determined in the cell-free culture supernatants (reflecting cellular necrosis) and cytoplasmic lysates of the cells (reflecting apoptosis) after 6 (**A**) and 24 h (**B**) of cell culturing. The concentration of curcumin (in µM) is given in the bars. The vehicle control was made with ethanol (0.2%). Data are means ± SEM of 4–8 independent experiments carried out in triplicate using cells from different donors, and are expressed in percent of untreated control (100%). Significant difference *vs*. untreated control: *P<0.05; **P<0.01.

By measurement of the internucleosomal DNA fragmentation rate in the cultured media and cell lysates, we determined whether the curcumin-induced decrease in RPE cell viability was mediated by inducing apoptosis and/or necrosis. An increased level of BrdU-labeled DNA fragments in the cell-free culture supernatants reflects cellular necrosis, while an increased level of BrdU-labeled DNA fragments in the cell lysates reflects apoptosis of the cells. Curcumin at higher concentrations (50 and 100 µM) induced a significant (*P*<0.05) increase in the DNA fragmentation rate in the cultured media. This effect was found at both 6 (Fig. 5A) and 24 h (Fig. 5B) after addition of curcumin to the culture media. An increase in the DNA fragmentation rate in the cell lysates was observed with curcumin at 100 µM after 6 h (Fig. 5A), and with curcumin at 10 and 50 µM after 24 h of stimulation (Fig. 5B). The data suggest that curcumin induces early necrosis and delayed apoptosis of RPE cells. In contrast to curcumin, triamcinolone acetonide up to 100 µM did not induce DNA fragmentation in RPE cells (not shown).

### Mechanisms of RPE Cell Death

**Figure 6:**
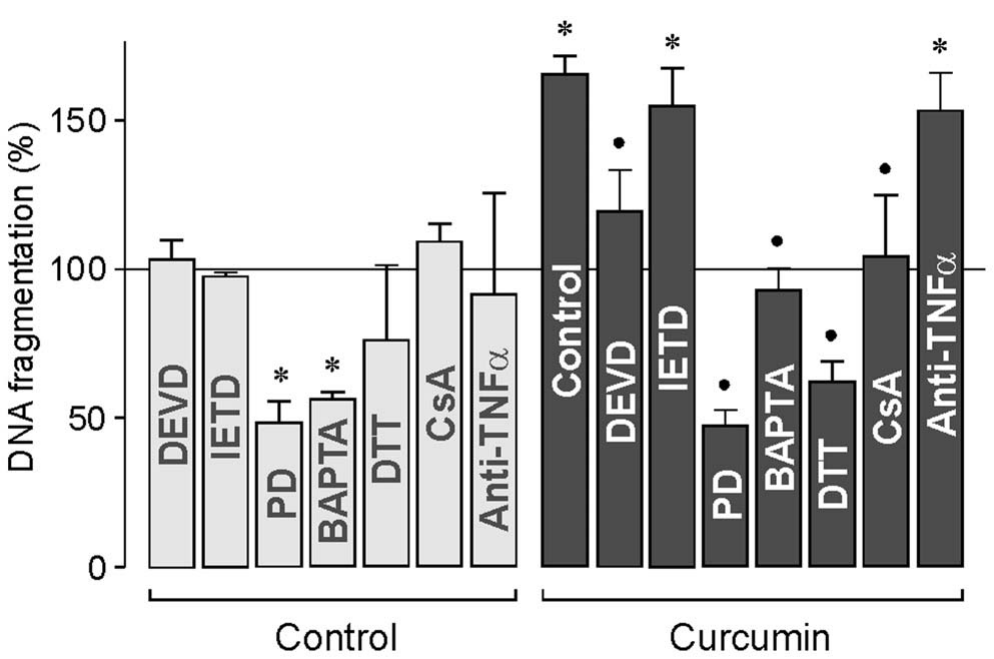
Mechanisms of curcumin-induced RPE cell apoptosis/necrosis. The rate of DNA fragmentation in the cultured media was determined after a 6 h stimulation of the cultures with curcumin (50 µM). The curcumin-induced DNA fragmentation was inhibited in the presence of the following agents: the caspase-3 inhibitor Ac-DEVD-CHO (DEVD; 100 µM), the calpain inhibitor PD150606 (PD; 100 µM), the cell-permeable calcium chelator BAPTA-AM (100 µM), the reducing agent dithiothreitol (DTT; 3 mM), and cyclosporin A (CsA; 1 µM), respectively. The curcumin-induced DNA fragmentation was not inhibited by the caspase-8 inhibitor Ac-IETD-CHO (IETD; 100 µM) and a neutralizing anti-TNFα antibody (20 µg/ml), respectively. Data are means ± SEM of 3–5 independent experiments carried out in triplicate using cells from different donors. Significant difference *vs*. untreated control: *P<0.05. Significant difference *vs*. curcumin control: ^•^ *P*<0.05.

To determine the mechanisms of the cytotoxic effect of curcumin, we tested various inhibitory agents. As shown in Figure 6, the inhibitor of the effector caspase-3, the tetrapeptide Ac-DEVD-CHO, reduced in part the curcumin-induced DNA fragmentation measured in the cultured media. The lack of effect of the caspase-3 inhibitor to completely block curcumin-induced DNA fragmentation may indicate the contribution of caspase-independent apoptotic and necrotic death pathways to the cytotoxic effect of the compound. An additional mechanism that may contribute to curcumin-induced cytotoxicity is the activation of the calcium-sensitive cysteine protease calpain. The calpain inhibitor PD150606 fully inhibited the curcumin-induced DNA fragmentation and decreased significantly (*P*<0.05) the DNA fragmentation rate measured under control conditions (Fig. 6). The inhibitory effect of the cell-permeable calcium chelator BAPTA-AM (Fig. 6) supports the assumption that intracellular calcium signaling is involved in mediating the cytotoxic effect of curcumin. The data suggest that both caspase-dependent and -independent mechanisms mediate curcumin-induced RPE cell death. Overproduction of reactive oxygen species was associated with activation of the mitochondrial apoptotic pathway [51]. As shown in Figure 6, the curcumin-induced DNA fragmentation measured in the cultured media was fully prevented in the presence of the cell-permeable dithiol-reducing agent dithiothreitol. One consequence of oxidative stress is activation of mitochondrial permeability transition which leads to mitochondrial dysfunction, energy failure, and enhanced free radical production. To determine whether mitochondrial membrane permeabilization is implicated in mediating the cytotoxic effect of curcumin, we tested the inhibitor of permeability transition, cyclosporin A [52]. As shown in Figure 6, cyclosporin A inhibited the curcumin-induced DNA fragmentation, but had no effect under control conditions. On the other hand, the curcumin-induced DNA fragmentation was not inhibited by a caspase-8 inhibitor, the tetrapeptide Ac-IETD-CHO (Fig. 6), as well as by a neutralizing anti-TNFα antibody (Fig. 6), suggesting that the cytotoxic effect of curcumin does not involve activation of extrinsic apoptotic pathways.

### Expression of Proapoptotic Proteins

**Figure 7:**
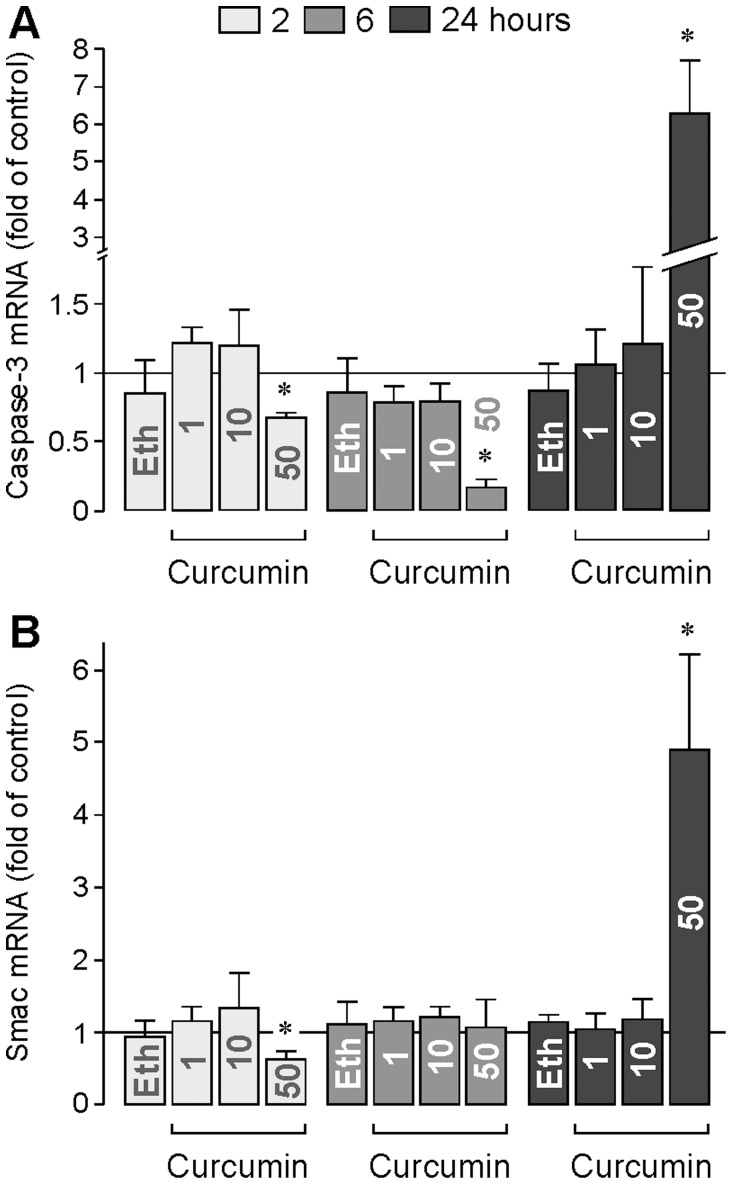
Effects of curcumin on the gene expression of caspase-3 (A) and Smac (B). The mRNA levels were determined by real-time RT-PCR after stimulation of the cells with curcumin for 2, 6, and 24 h, respectively. The concentrations of curcumin (in µM) are given in the bars. The vehicle control was made with ethanol (Eth; 0.2%). Data are means ± SEM of 4 independent experiments carried out in triplicate using cells from different donors. Significant difference *vs*. untreated control: *P<0.05.

Inhibition of caspase-3 activation partially prevented the cytotoxic effect of curcumin (Fig. 6). Thus, we determined whether curcumin induces (in addition to activation of caspase-3) also alterations in the gene expression of caspase-3. As shown in Figure 7A, curcumin at 50 µM induced an early downregulation (after 2 and 6 h of stimulation) and a delayed upregulation of caspase-3 (after 24 h of stimulation). A similar regulation was found in the gene expression of the proapoptotic protein Smac which binds to inhibitor of apoptosis proteins and deactivates them. As shown in Figure 7B, curcumin at 50 µM induced an early downregulation (after 2 h of stimulation) and a delayed upregulation of Smac (after 24 h of stimulation). On the other hand, curcumin (up to 50 µM) did not alter the gene expression of caspase-8 and Bax (not shown).

### Activation of Intracellular Signal Transduction Molecules

**Figure 8:**
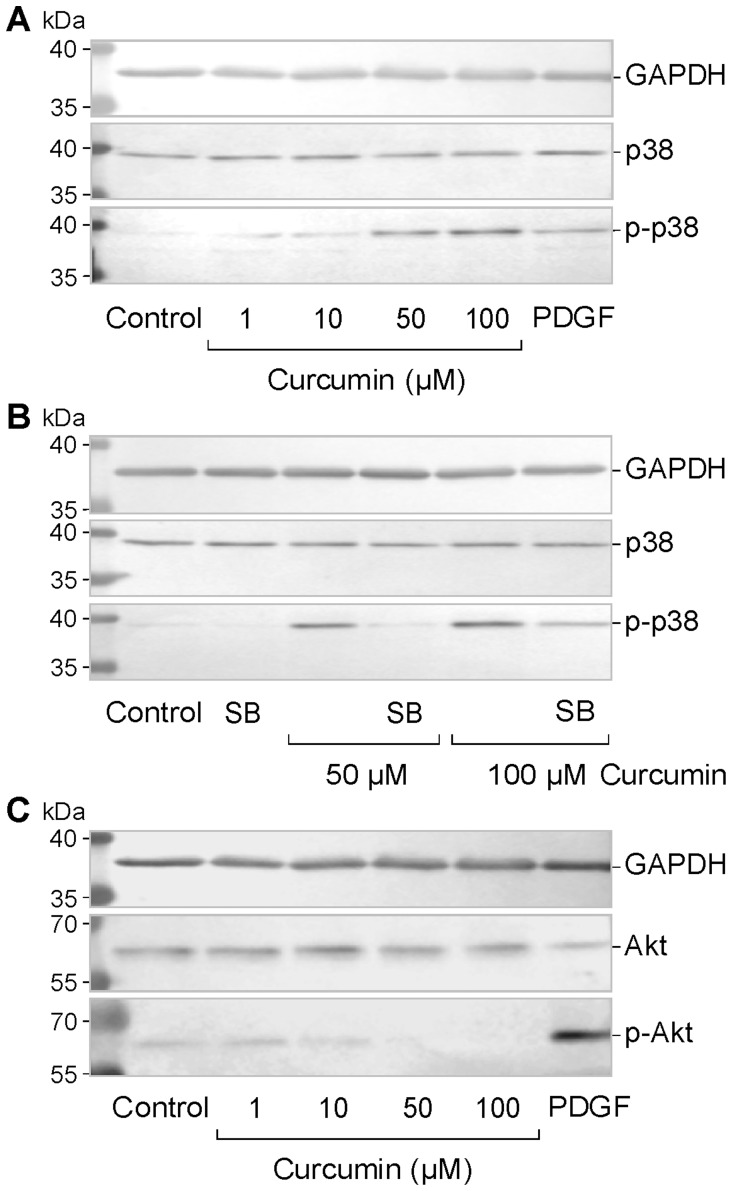
Curcumin induces phosphorylation of p38 MAPK and decreases phosphorylation of Akt in RPE cells. **A.** Dose-dependence of the curcumin effect on the phosphorylation level of p38 MAPK. As control, PDGF (10 ng/ml) was tested. **B.** The inhibitor of p38 MAPK activation, SB203580 (SB; 10 µM), decreased the curcumin-induced phosphorylation of p38 MAPK. Curcumin was tested at 50 and 100 µM. **C.** Dose-dependence of the curcumin effect on the phosphorylation level of Akt. The cultures were stimulated with the agents for 15 min. The amounts of total proteins are shown above; the amounts of phosphorylated proteins are shown below. Similar results were obtained in 3 independent experiments using cells from different donors.

We used Western blot analysis to determine whether curcumin activates key molecules of intracellular signal transduction cascades. We found that curcumin (up to 100 µM; 15 min) did not alter the phosphorylation levels of ERK1/2 and SAPK/JNK in RPE cells (data not shown). However, curcumin induced dose-dependently phosphorylation of p38 MAPK protein (Fig. 8A). The curcumin-induced phosphorylation of p38 MAPK was inhibited by the selective inhibitor of p38 MAPK activation, SB203580 (Fig. 8B). In addition, curcumin induced a dose-dependent decrease in the phosphorylation level of protein kinase B (Akt) in RPE cells (Fig. 8C).

**Figure 9:**
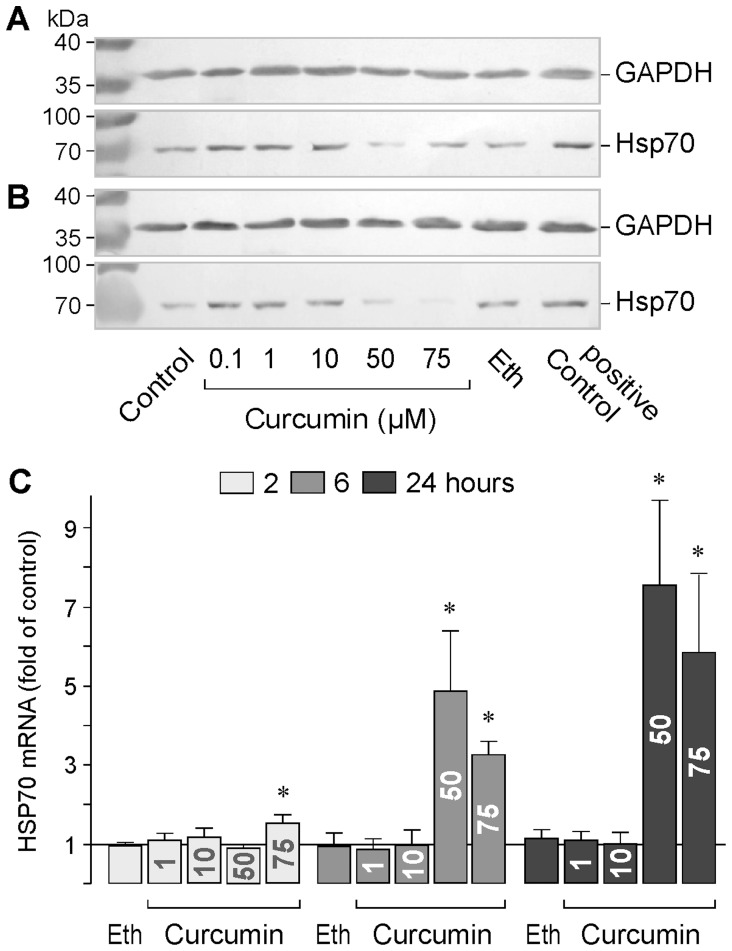
Effects of curcumin on the protein level (A, B) and mRNA expression (C) of HSP70 in RPE cells. The effect on the protein level was determined by Western blotting after a 6 h (**A**) and a 24 h (**B**) stimulation of the cells with curcumin. Similar results were obtained in 3 independent experiments using cells from different donors. In **A** and **B**, untreated cells were used as control; the positive control was made by incubation of the cells at 42°C for 1 h followed by incubation at 37°C for 3 h. The vehicle control was made with ethanol (Eth; 0.2%). The gene expression level (**C**) was determined with real-time RT-PCR after stimulation of the cells with curcumin for 2, 6, and 24 h, respectively. Data are means ± SEM of 3–4 independent experiments carried out in triplicate using cells from different donors. Significant difference *vs*. untreated control: *P<0.05.

Expression of HSP70 HSP70 is a known prosurvival protein, e.g., in tumor cells [53]. By repairing protein damage and preventing protein aggregations, HSPs also protect RPE cells from harmful effects of stress stimuli [54]. We determined whether curcumin induces alterations in the protein level and gene expression of HSP70 in RPE cells. As shown in Figures 9A and B, incubation of the cells at 42°C for 1 h induced an increase in the HSP70 protein content of the RPE cells as compared to untreated control. Curcumin at lower concentrations (0.1 and 1 µM) induced also an increase in the HSP70 protein level in RPE cells, whereas curcumin at higher concentrations (50 and 75 µM) induced a decrease in the cellular HSP70 protein level (Fig. 9A, B). On the other hand, the HSP70 mRNA level was increased after a 6- and 24 h stimulation of the cells with curcumin at higher concentrations (Fig. 9C).

## Discussion

The clinical interest in curcumin was motivated by data obtained in *in-vitro* and *in-vivo* studies that showed that curcumin induces proliferation arrest and apoptotic and necrotic death in a variety of tumor cells [12], [55], [56]. Curcumin is also suggested to be helpful as concomitant therapy of diseases associated with chronic inflammation, and may be used as adjuvant immunosuppressant [1], [8], [9]. Based upon data obtained in animal models of retinopathies and cultured retinal cells, it has been suggested that curcumin may also have potential benefits in inhibiting the development and progression of various blinding retinal disorders including diabetic retinopathy, age-related macular degeneration, and retinitis pigmentosa [28]–[30]. It has been shown that curcumin increases the survival of retinal cells under various *in-vivo* and *in-vitro* conditions including animal models of diabetic retinopathy, light-induced retinal degeneration, retinitis pigmentosa, and ischemia-reperfusion injury of the retina [28]–[32], [34]. However, it was also shown that curcumin induces apoptosis in human retinal endothelial cells [35] and decreases the viability of RPE cells [46]. In the present study, we show that curcumin has toxic effects on human RPE cells. It induces early necrosis and delayed apoptosis in RPE cells which are mediated by caspase-dependent and -independent mechanisms, via intrinsic and mitochondrial apoptotic pathways. In addition, curcumin alters the expression and secretion of angiogenic cytokines in RPE cells. The cytotoxic effects of curcumin were observed at doses described to be effective in the treatment of tumor cells. Curcumin was shown to inhibit the proliferation and to induce death of cancer cells at concentrations between 5 and 50 µM, after incubation for several hours [18], [57]–[60]. Antiviral effects were found at much higher concentrations of curcumin (≥100 µM) [6]. In the present study, we found that curcumin at 10 µM induced apoptosis of RPE cells (Fig. 5B) while higher concentrations induced necrosis of the cells (Fig. 5A, B). The present data confirm data of a previous study that showed pro-apoptotic effects of curcumin at 10 µM in RPE cells [46]. Curcumin at 10 µM was also shown to induce apoptosis of retinal endothelial cells [35].

Curcumin reduces the expression (Fig. 1A) and secretion (Fig. 1B) of VEGF from RPE cells. It has been shown in animal models of diabetic retinopathy that dietary curcumin inhibits the increase in retinal VEGF [28], [31]. The present data suggest that the downregulation of VEGF is mediated by a direct action of curcumin on retinal cells. Though downregulation of VEGF may have beneficial effects in the treatment of diabetic retinopathy and choroidal neovascularization, systemic administration of curcumin in other clinical settings, e.g., in the treatment of cancer, may have detrimental effects because VEGF is also constitutively released from the RPE under normal conditions; the constitutively released VEGF is critical in the maintenance of the healthy choriocapillaris [42]. The closure of choriocapillaris endothelial cell fenestrations induced by a reduction of the constitutively released VEGF [42], [61] may contribute to hypoxic conditions in the outer retina which is a key feature of the wet form of age-related macular degeneration [37]. Significant inhibition of hypoxia-induced secretion of VEGF from RPE cells was found with curcumin at 10 µM (Fig. 1B). Because curcumin at this concentration induces delayed apoptosis in RPE cells (Fig. 5A, B), the harmful effects of downregulation of VEGF on the choriocapillaris may be accompanied by curcumin-induced degeneration of the RPE. Furthermore, the stimulatory effects of curcumin at low concentrations (1 and 10 µM) on the expression of bFGF (Fig. 2A) and HGF (Fig. 2B), on the secretion of bFGF (Fig. 2C), and on the proliferation of the cells (Fig. 3A), may support the development of neovascular and proliferative retinal diseases. We found that curcumin at 10 µM increased the expression of bFGF and HGF after 2 h of stimulation, but decreased the expression of the factors after 6 h of stimulation (Fig. 2A, B). The reason of the different effects of curcumin is unclear and might be related to the time-dependent activation of signaling cascades that lead to cellular necrosis.

The apoptotic death of RPE cells, followed by photoreceptor cell death, is a major factor contributing to the pathogenesis of the dry form of age-related macular degeneration [38]. In the present study, we show that curcumin decreased dose- and time-dependently the viability of RPE cells via induction of early necrosis (at concentrations above 10 µM; Fig. 5A) and delayed apoptosis (at concentrations above 1 µM; Fig. 5B). Thus, it can not be ruled out that long-term intake of curcumin may facilitate the development of age-related retinal diseases. We found that the cytotoxic effect of curcumin was mediated by various mechanisms including activation of caspase-3 and calpain, intracellular calcium signaling, mitochondrial permeability, oxidative stress, and increased phosphorylation of p38 MAPK and decreased phosphorylation of Akt protein. The partial inhibitory effect of the caspase-3 inhibitor DEVD on the curcumin-induced DNA fragmentation (Fig. 6) suggests that (in addition to caspase-dependent pathways) caspase-independent apoptotic pathways and necrotic pathways are involved in the toxic effect of curcumin. The lack of effects of the caspase-8 inhibitor IETD and the neutralizing anti-TNFα antibody on the curcumin-induced DNA fragmentation (Fig. 6) largely rules out the possibility that activation of extrinsic apoptotic pathways plays an important role in curcumin-induced cytotoxicity. The present data confirm and extent data of a recent study which showed that curcumin induces caspase-3/7-dependent but caspase-8-independent death and necrosis of RPE cells [46]. The cytotoxic effect of curcumin is also mediated by calpain activation, likely due to intracellular calcium overload (Fig. 6). Activation of calpain by calcium is known to trigger caspase-dependent and -independent cell death pathways. Mitochondrial apoptosis is triggered by intracellular stimuli such as calcium overload and reactive oxygen species [51]. We found that both mitochondrial permeability transition and oxidative stress are mediators of the cytotoxic effect of curcumin (Fig. 6). We found also that curcumin induces activation of p38 MAPK (Fig. 8A, B) and inhibition of Akt (Fig. 8C). p38 MAPK is generally considered to be a proapoptotic MAPK, whereas activation of the phosphatidylinositol-3 kinase-Akt signaling pathway was shown to suppress apoptosis and to promote cell survival in various different cell systems [62]–[64] including RPE cells [65]. Curcumin induced an early downregulation and a delayed upregulation of the proapoptotic proteins caspase-3 (Fig. 7A) and Smac (Fig. 7B). The functional significance of the downregulation of these proteins is unknown. It may be speculated that the curcumin-induced downregulation of proapoptotic proteins may inhibit early apoptosis whereas upregulation of caspase-3 (Fig. 7A) and Smac (Fig. 7B) after 24 h of stimulation may contribute to the curcumin-induced delayed apoptosis of RPE cells observed in the DNA fragmentation experiments (Fig. 5B). We found that generation of oxidative stress contributes to the cell viability-reducing effect of curcumin (Fig. 6). It has been shown that reactive oxygen species such as hydroxyl radicals or superoxide can cause direct damage to lipid membranes, resulting in necrotic death of the cells. However, further research is required to determine the relative contribution of different biochemical cascades leading to curcumin-induced RPE cell death.

We found that curcumin at higher concentrations induced a decrease in the HSP70 protein level in RPE cells (Fig. 9A, B) whereas the HSP70 mRNA level was increased (Fig. 9C). HSP70 is an anti-apoptotic protein [53]. By functioning as molecular chaperones, HSPs are involved in the protection of RPE cells from harmful stimuli [54]. Our data are in agreement with a recent study that showed that the cytotoxic effects of curcumin are in part mediated by inducing a decrease in the cellular content of HSP70 [66]. Another mechanism that may contribute to the cytoprotective effect of HSP70 following cellular stress is the posttranscriptional regulation of the gene expression [67]. It has been recently shown that HSP70 recognizes and stabilizes selected mRNAs, e.g., VEGF mRNA [67]. It remains to be determined in future experiments whether the inhibitory effects of curcumin at higher concentrations on the expression of growth factors (Fig. 2A, B) including VEGF (Fig. 1A) are (at least in part) mediated by a decrease in the cellular HSP70 level.

It has been suggested that curcumin is a promising drug for the treatment of cancer and may have also benefits in the treatment of retinal diseases. However, further assessment for potential hazards of curcumin should be considered before the compound is broadly used in the clinical setting [18]. The cytotoxic effect of curcumin found in the present study is mediated by pathogenic mechanisms which play also a role in the development of retinal diseases; for example, oxidative stress-induced mitochondrial dysfunction in RPE cells is an important pathogenic factor of age-related macular degeneration [68]. It is suggested that, during the intake of curcumin as concomitant therapy of cancer, as antiviral treatment, or in the treatment of eye diseases, retinal function should be monitored carefully.
